# Percutaneous endoscopic gastrostomy versus fluoroscopic gastrostomy in amyotrophic lateral sclerosis (ALS) sufferers with nutritional impairment: A meta-analysis of current studies

**DOI:** 10.18632/oncotarget.22288

**Published:** 2017-11-06

**Authors:** Biying Yang, Xiaolei Shi

**Affiliations:** ^1^ Guangdong Provincial Hospital of Chinese Medicine, The Second Affiliated Hospital of Guangzhou University of Chinese Medicine, Guangzhou, Guangdong, P.R. China; ^2^ Department of Neurology, The First Affiliated Hospital, Yijishan Hospital of Wannan Medical College, Wuhu, Anhui, P.R. China; ^3^ Kinsmen Laboratory of Neurological Research, University of British Columbia, Vancouver, BC, Canada

**Keywords:** amyotrophic lateral sclerosis (ALS), gastrostomy, meta-analysis

## Abstract

Gastrostomy is recommended for Amyotrophic Lateral Sclerosis (ALS) patients with malnutrition. There are two main methods of gastrostomy insertion: Percutaneous Endoscopic Gastrostomy (PEG) and Fluoroscopic Gastrostomy (FG). The latter included Radiologically Inserted Gastrostomy (RIG) and Per-oral Image-Guided Gastrostomy (PRG). A meta-analysis was conducted to compare these approaches in terms of survival outcomes, pain occurrence and success rate, through the literature search in PubMed, Web of Science and Cochrane Library. A total of 7 studies with 701 cases (322 in PEG, 264 in RIG and 115 in PRG) were enrolled in the final analysis. The lack of differences between the comparisons (PEG vs. PRG, PEG vs. RIG and PEG vs. PRG+RIG) on 30-day mortality and survival length was confirmed. For the pooling analysis of peri- and post-procedural complications, patients with PEG had a lower incidence of pain than cases with PRG and RIG together (*P* < 0.001). The same trends could be found when compared with PRG and RIG, separately (*P* < 0.05 and *P* < 0.001, respectively). And PEG showed a lower rate of successful attempts than PEG and RIG (*P* < 0.05). For other complications, we didn't find any differences. This meta-analysis demonstrates that PEG, PRG and RIG had their intrinsic advantages. The current evidences could not determine the preference of them. Further investigations should be done to reveal the most appropriate method for ALS patients.

## INTRODUCTION

Amyotrophic Lateral Sclerosis (ALS) is the most common form of motor neuron disease in adults [1]. During the course of this disorder, malnutrition is common and act as an independent prognostic factor for survival in patients with ALS. It could be attributed to a series of progressive factors, including anorexia, dysphagia, chewing difficulty and psychological upset [2]. Approaches that maintaining a neutral nutritional balance, including altering food consistency, increasing the number of daily meals and use of feeding assistance devices, prevent malnutrition and improve physical functioning, quality of life and survival. However, as dysphagia gradually progresses, enteral feeding is often suggested for nutritional management.

Gastrostomy enteralfeeding is an effective assistance intervention approach to maintain adequate nutritional intake among these patients. The American Academy of Neurologists (AAN) and European Federation of Neurological Societies (EFNS) guidelines both recommended gastrostomy for ALS patients with nutritional deficits [3, 4]. It is believed that gastrostomy feeding tube should be placed before respiratory insufficiency develops to improve survival and quality of life. There are two main methods of gastrostomy tube insertion: endoscopy and fluorescence assistedmethods, including Percutaneous Endoscopic Gastrostomy (PEG), Radiologically Inserted Gastrostomy (RIG) and Per-oral Image-Guided Gastrostomy (PRG) [5]. The two main methods are both widely used in the clinical practice for their improvement on survival status. However, studies comparing them didn't have a consistent opinion on the tube insertion method preference. Some studies believed that ALS survival status was not correlated with the method selection [6, 7], while some researchers found that PRG should be encouraged for a longer survival [8, 9]. Also the complications that might occurred during and after operation, should be taken into consideration in the clinical selection. For example, PEG is often challenged for a lower insertion success rate and a higher incidence of aspiration [8]. But PEG is also to be found to have a lower rate of complications, including pain and tube replacement [10]. These controversies might hinder the application among the physicians. To solve the discrepancies, we then take this meta-analysis to demonstrate it.

## RESULTS

### Flow of included studies

**Figure 1:**
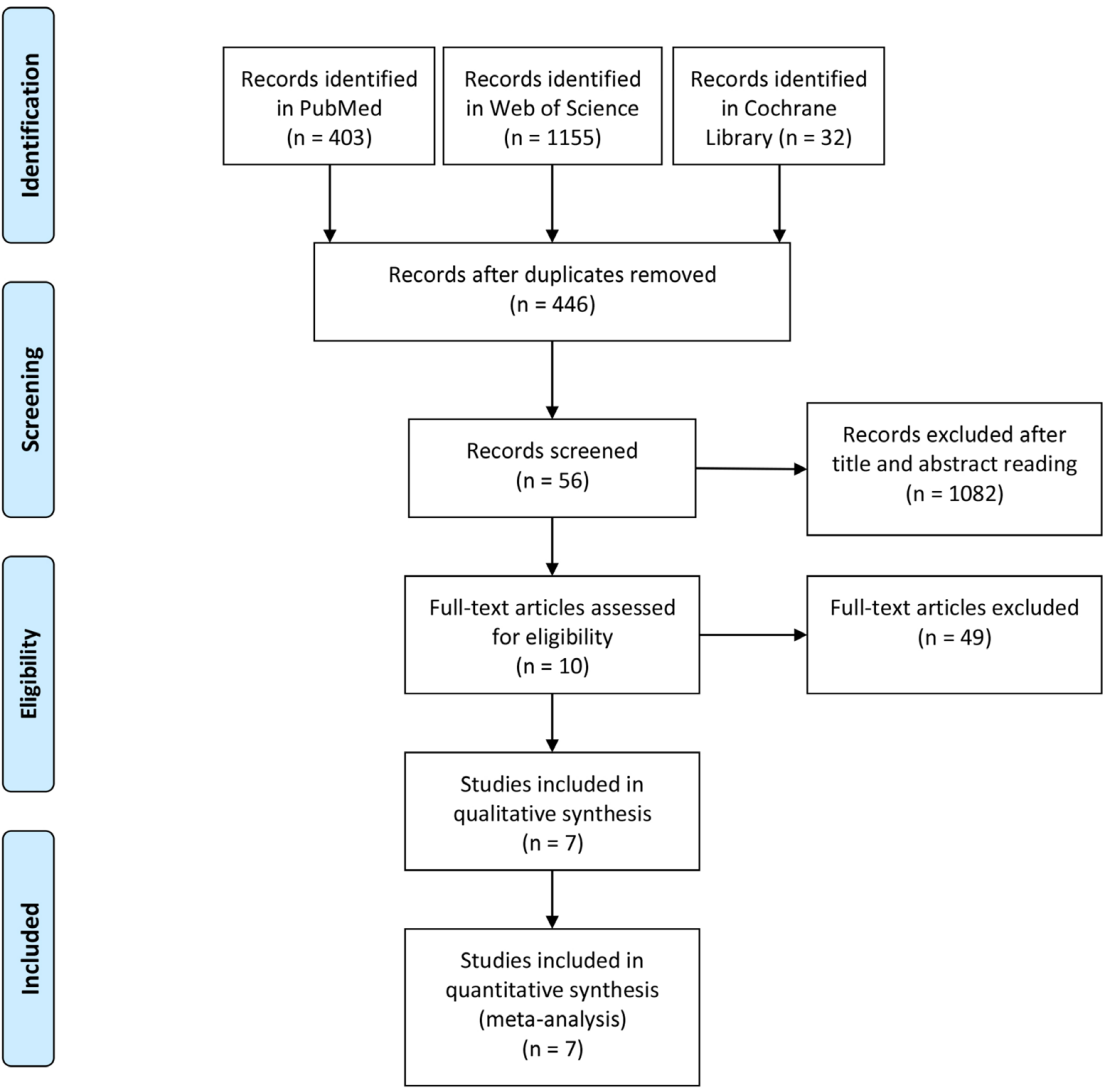
Flow diagram of the meta-analysis

The flow diagram was shown in Figure 1, according to the Quality of Reporting of Meta-analyses-statements with the total number of citations retrieved by the search strategy and the number included in this study. In total, 1590 articles from PubMed, Web of Science and Cochrane Library were found. After excluding the duplicates, 1144 studies were found. After further exclusion and full text assessment,7 studies, including 701 cases (322 in PEG, 264 in RIG and 115 in PRG) fulfilling the predefined inclusion criteria, were enrolled in the final analysis (Figure 1). All publications were full-text articles. Agreement between the two reviewers was 97% for study selection and 93% for quality assessment of trials.

### Study characteristics and quality assessment

The baseline characteristics were shown in Table 1. Among all the studies included, there were 5 retrospective and 2 prospective studies. 3 studies [8, 9, 11] compared PEG and PRG methods; 3 studies [6, 10, 12] examined PEG and RIG procedures; and 1 study [5] assessed PEG, RIG and PRG interventions. All studies clarified the study period, and two of them indicated the follow-up time. All the cases included were from Caucasian population, with 6 studies in Europe and 1 study in North America. For quality assessment, we found that five studies scored 6 points or more, and one has a relatively large sample size.

**Table 1:** Baseline characteristics of the included studies

Study	Year	Design	Time	Period	Geography	Group	No.	Gender (M)	Age (y)^*^	Quality
Thornton [8]	2002	Retro	NA	1997.07–2001.03	Ireland	PEG	11	NA	NA	7
					PRG	25	NA	NA		
Chio [9]	2004	Retro	2 m	2000.10–2002.12	Italy	PEG	25	13	65.1 ± 10.3	7
					PRG	25	12	68.9 ± 9.5		
Desport [6]	2005	Pros	NA	1999.03–2002.11	France	PEG	30	12	65.7 ± 10.3	6
					RIG	20	4	66.1 ± 9.7		
Shaw [12]	2006	Retro	NA	1998.11–2003.11	UK	PEG	18	9	62 (26–85)	5
						RIG	72	38	60 (31–86)	
Blondet [7]	2010	Retro	NA	1999–2005	France	PEG	18	6	66.2 ± 11.2	3
						PRG	22	11	66 ± 12	
Allen [10]	2013	Retro	NA	2009.01–2012.03	USA	PEG	57	35	59.7 ± 11.6	8
					RIG	51	29	59.0 ± 11.3		
						PEG	163	90	64.2 ± 11.7	9
						RIG	121	62	63.6 ± 9.8	
ProGas Study Group [5]	2015	Pros	12 m	2010.11–2014.01	UK	PRG	43	25	67.2 ± 12.6	

y, year; Retro, retrospective; Pros, prospective; NA, not applicable; m, month; M, male; PEG, Percutaneous Endoscopic Gastrostomy; PRG, Per-oral Image-Guided Gastrostomy; RIG, Radiologically Inserted Gastrostomy.

### Survival analysis

**Figure 2:**
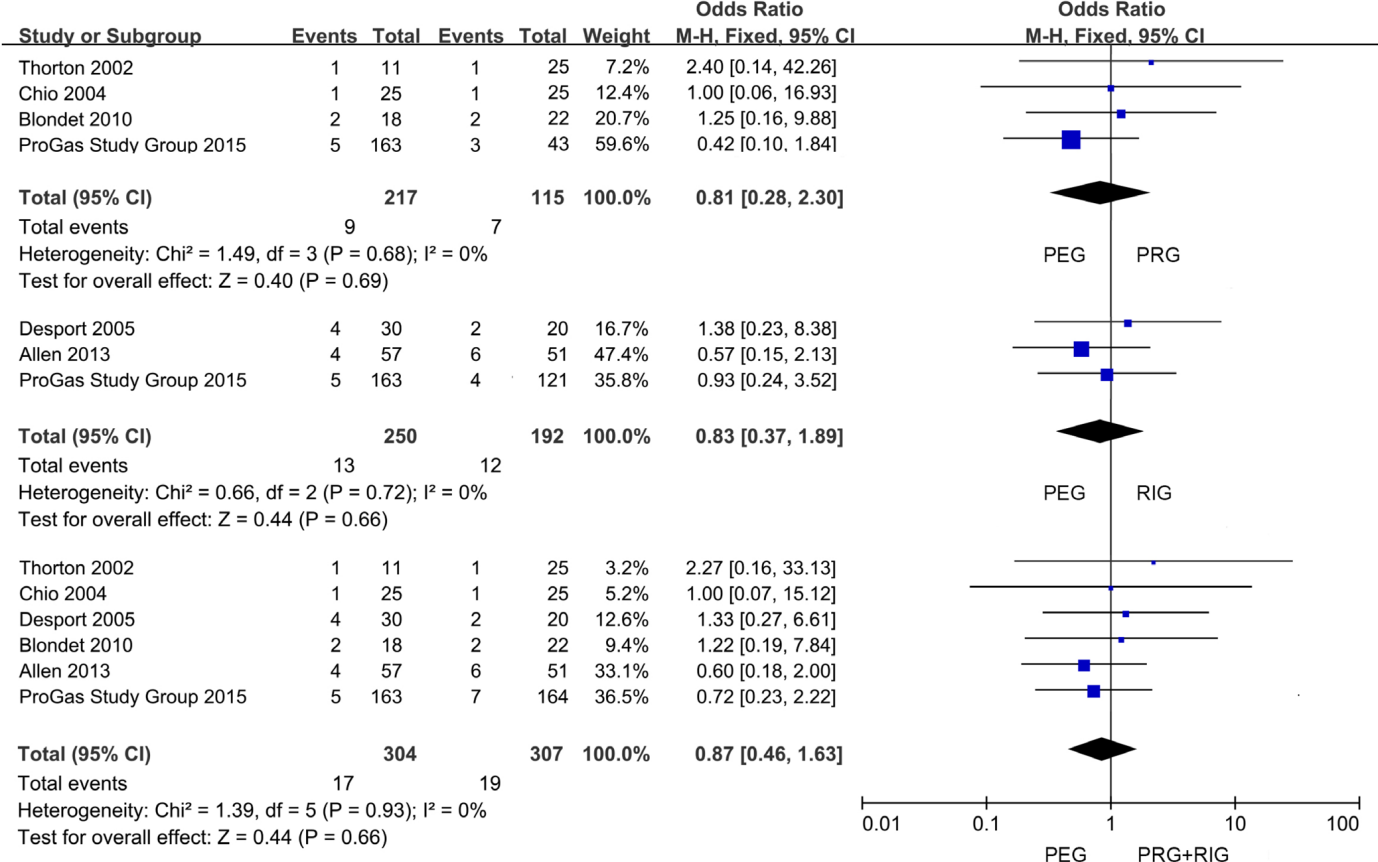
Forest plot and meta-analysis of 30-day mortality

**Figure 3:**
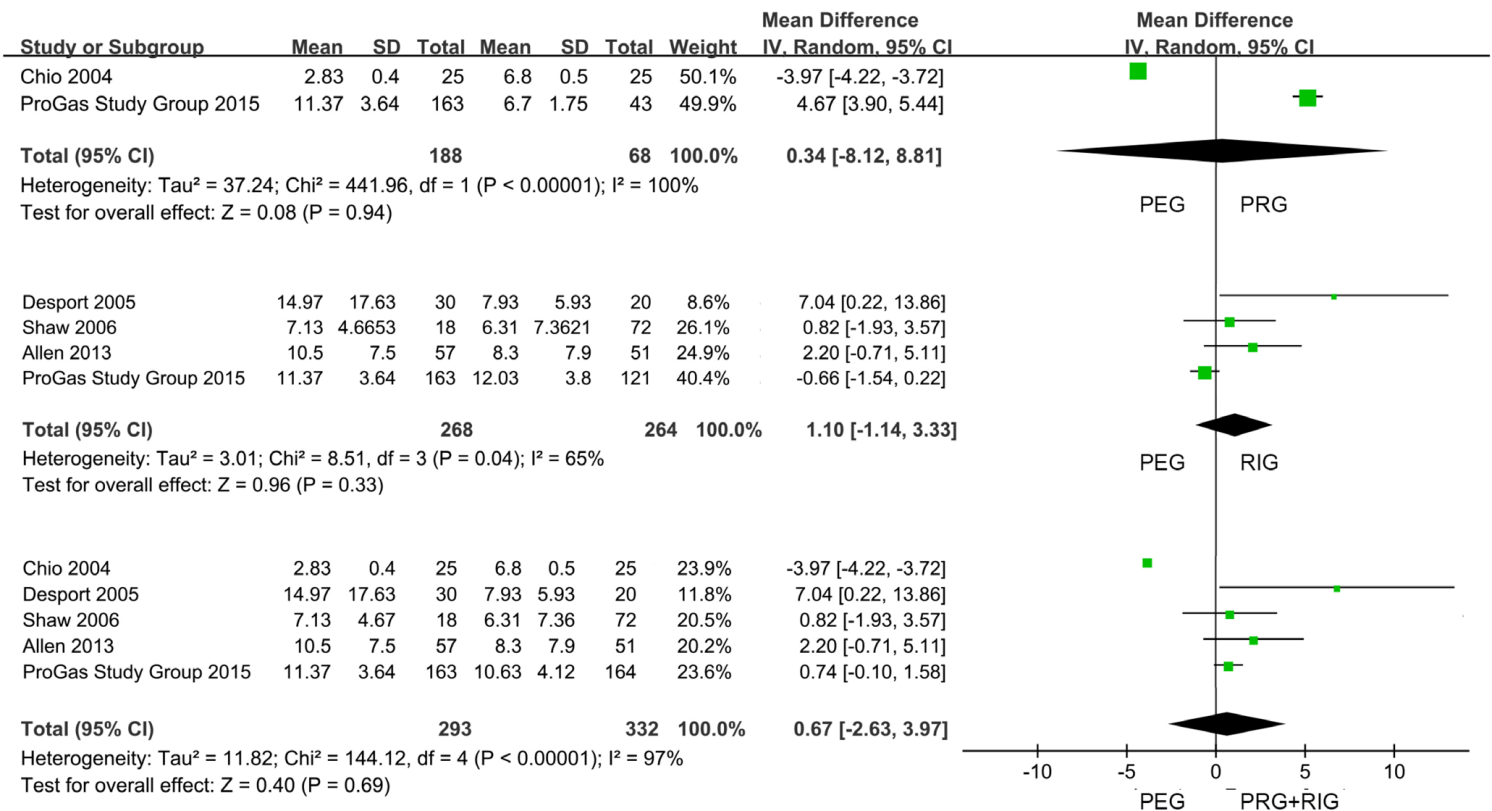
Forest plot and meta-analysis of survival length

The data of 30-day mortality and survival length were shown in Table 2. 6 studies displayed 30-day mortality data after placement, and only 1 study gave 6-month mortality information. All the studies provided the survival time records after operation. But 2 of them only showed the mean or median survival time; the rest depicted as Mean ± SD, Median (95% CI), Median (25th IQR). We first performed a meta-analysis of the survival data (Table 3). Compared with FG (the combination of PRG and RIG) in 6 studies, PEG had no effect on 30-day mortality of the sufferers (PEG versus PRG+RIG: 5.59% versus 6.19%; OR, 0.87; 95% CI, 0.46–1.63; *P* = 0.66) (Figure 2). In further analysis, we found from the pooling data of 4 studies which compared PEG and PRG, that there was no significance on 30-day mortality (PEG versus PRG: 4.15% versus 6.09%; OR, 0.81; 95% CI, 0.28–2.30; *P* = 0.69) (Figure 2). Also, no significant difference was found between PEG and RIG from the meta-analysis of 30-day mortality data from 3 studies (PEG versus RIG: 5.20% versus 6.25%; OR, 0.83; 95% CI, 0.37–1.89; *P* = 0.66) (Figure 2). Data revealed that no marked differences of survival time were found in the comparison of patients with PEG and cases with PRG and RIG together (PEG versus PRG+RIG: WMD, 0.67; 95% CI, –2.63–3.97; *P* = 0.69) (Figure 3). Subgroup analysis also found no obvious significance in the two comparisons (PEG versus PRG: WMD, 0.34; 95% CI, –8.12–8.81; *P* = 0.94; PEG versus RIG: WMD, 1.10; 95% CI, –1.14–3.33; *P* = 0.33) (Figure 3).

**Table 2:** Clinical outcomes of all the studies

Study	Group	30-day mortality	Survival time
Thornton	PEG	1	11.2m^a^
	PRG	1	9.5m^a^
Chio	PEG	1	(85 ± 12)d^c^
	PRG	1	(204 ± 15)d^c^
Desport	PEG	4	(449 ± 529)d^c^
	RIG	2	(238 ± 178)d^c^
Shaw	PEG	NA	7.13 (4.81–9.45)m^d^
	RIG	NA	6.31 (4.58–8.04)m^d^
Blondet	PEG	2	302d^b^
	PRG	2	191d^b^
Allen	PEG	4	(10.5 ± 7.5)m^c^
	RIG	6	(8.3 ± 7.9)m^c^
ProGas	PEG	5	341 (164)d^e^
Study	RIG	4	361 (171)d^e^
Group	PRG	3	201 (116)d^e^

PEG, Percutaneous Endoscopic Gastrostomy; PRG, Per-oral Image-Guided Gastrostomy; RIG, Radiologically Inserted Gastrostomy; NA, Not Applicable; m, month; d, day. Survival length was depicted as Mean^a^, Median^b^, Mean ± SD^c^, Median (95% CI)^d^, Median (25th IQR)^e^; ^f^means the incidence number in total events.

**Table 3:** Meta-analysis results of all the studies

				Study Heterogeneity			
Outcome of interests	Study number	OR/WMD (95% CI)	*P* value	χ^2^	df	*I*^2^, %	*P* value
***Survival***							
**30-day mortality**							
PEG vs. PRG	4	0.81 (0.28, 2.30)	0.69	1.49	3	0	0.68
PEG vs. RIG	3	0.83 (0.37, 1.89)	0.66	0.66	2	0	0.72
PEG vs. PRG+RIG	6	0.87 (0.46, 1.63)	0.66	1.39	5	0	0.93
**Survival length**							
PEG vs. PRG	2	0.34 (–8.12, 8.81)	0.94	441.96	1	100	<0.00001
PEG vs. RIG	4	1.10 (–1.14, 3.33)	0.33	8.51	3	65	0.04
PEG vs. PRG+RIG	5	0.67 (–2.63, 3.97)	0.69	144.12	4	97	<0.00001
***Peri-complication***							
**Difficult procedure**							
PEG vs. PRG	2	1.20 (0.58, 2.51)	0.62	0.55	1	0	0.46
PEG vs. RIG	2	1.59 (0.81, 3.16)	0.18	0.01	1	0	0.91
PEG vs. PRG+RIG	3	1.45 (0.84, 2.51)	0.18	0.15	2	0	0.93
**Success Attempt Rate**							
PEG vs. PRG	4	0.19 (0.03, 1.33)	0.09	7.18	3	58	0.07
PEG vs. RIG	2	0.38 (0.04, 3.76)	0.41	3.43	1	71	0.06
PEG vs. PRG+RIG	5	0.18 (0.04, 0.86)	0.03	10.11	4	60	0.04
**O_2_ desaturation**							
PEG vs. RIG	2	2.13 (0.56, 8.17)	0.27	0.02	1	0	0.88
PEG vs. PRG+RIG	2	1.32 (0.45, 3.88)	0.61	0.08	1	0	0.77
**Distress**							
PEG vs. RIG	2	1.92 (0.12, 30.63)	0.65	2.83	1	65	0.09
PEG vs. PRG+RIG	2	1.87 (0.13, 26.48)	0.64	2.58	1	64	0.10
**Respiratory arrest**							
PEG vs. PRG	2	7.29 (0.27, 193.68)	0.24	NA	NA	NA	NA
PEG vs. RIG	2	3.77 (0.41, 34.92)	0.24	NA	NA	NA	NA
PEG vs. PRG+RIG	3	4.55 (0.71, 29.04)	0.11	0.11	1	0	0.74
***Post-complication***							
**Infection**							
PEG vs. PRG	2	2.20 (0.67, 7.19)	0.19	0.58	1	0	0.45
PEG vs. PRG+RIG	2	0.90 (0.49, 1.67)	0.74	1.65	1	39	0.20
**Pain**							
PEG vs. PRG	2	0.49 (0.24, 0.98)	0.04	0.83	1	0	0.36
PEG vs. RIG	3	0.42 (0.25, 0.69)	0.0007	1.69	2	0	0.43
PEG vs. PRG+RIG	4	0.42 (0.27, 0.67)	0.0002	2.24	3	0	0.52

OR, odds ratio; WMD, weighted mean difference; PEG, Percutaneous Endoscopic Gastrostomy; PRG, Per-oral Image-Guided Gastrostomy; RIG, Radiologically Inserted Gastrostomy.

### Complication analysis

**Figure 5:**
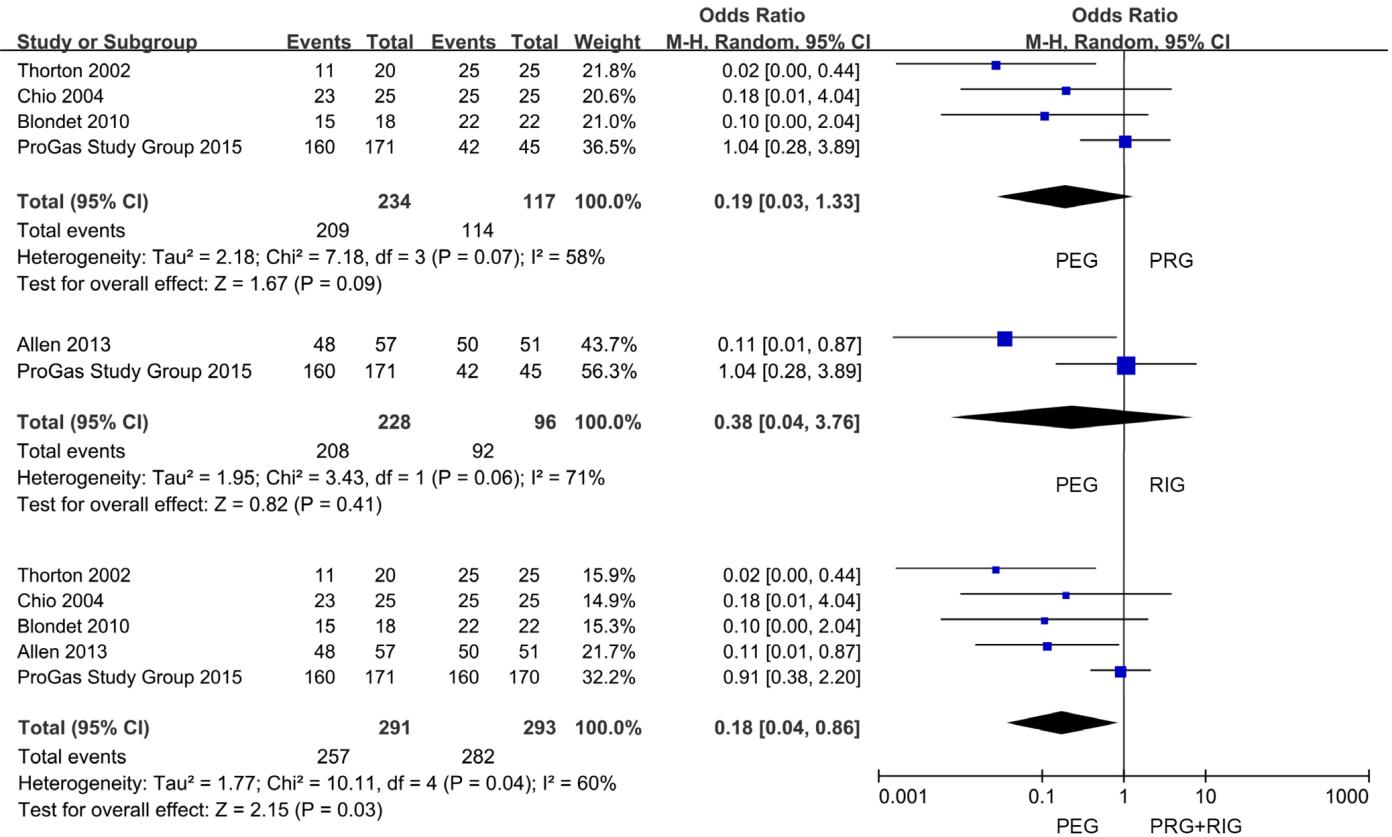
Forest plot and meta-analysis of success gastrostomy attempt rate

We supplied the detailed information of peri- and post-procedural complications in Supplementary Tables 1 and 2. The complications without clear and definite incidence information were marked as not applicable (NA). In the peri-complications (Table 3), we made the pooling analysis of difficult procedure, success attempt, O_2_ desaturation, distress and respiratory arrest. And we failed to assess laryngeal spasm and harmorrhage for the lack of sufficient data. The findings showed that there were no differences among all the comparisons in difficult procedure (Supplementary Figure 1), O_2_ desaturation (Supplementary Figure 2), distress (Supplementary Figure 3) and respiratory arrest (Supplementary Figure 4) (*P* > 0.05). However, we found that PEG operation had a lower success attempt rate than PRG and RIG together (PEG versus PRG+RIG: 88.32% versus 96.25%; OR, 0.18; 95% CI, 0.04-0.86; *P* < 0.05) (Figure 5). Whereas, there were no differences in the comparison of PEG with PRG and RIG separately (PEG versus PRG: 89.32% versus 97.44%; OR, 0.19; 95% CI, 0.03-1.33; *P* > 0.05; PEG versus RIG: 91.23% versus 95.83%; OR, 0.38; 95% CI, 0.04-3.76; *P* ﹥ 0.1) (Figure 5).

**Figure 4:**
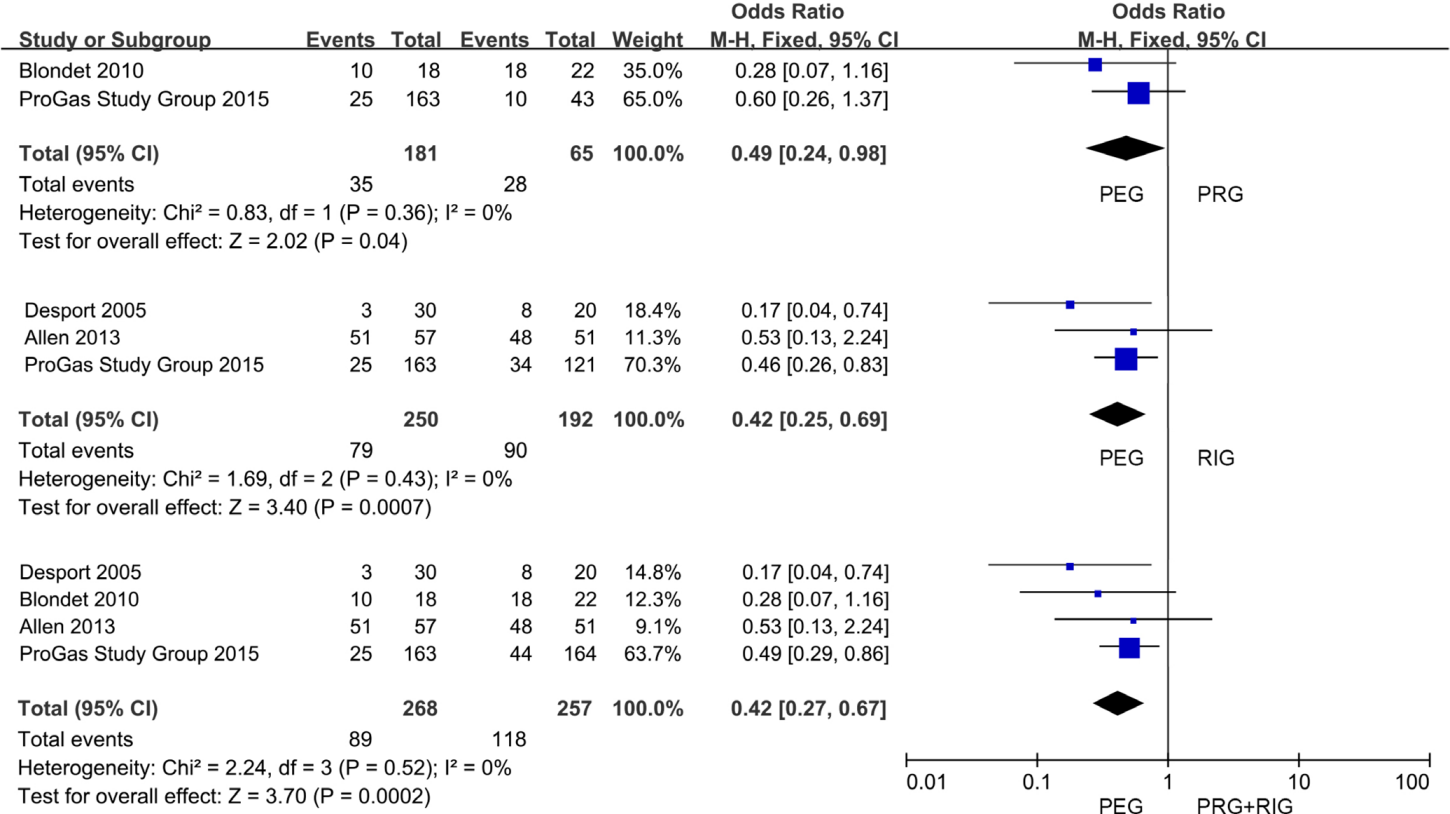
Forest plot and meta-analysis of pain occurrence

We then focused on the post-procedural complications (Supplementary Table 2). Only infection and pain were analyzed with enough information. We found no differences in the comparison of infection occurrence (*P* > 0.05) (Supplementary Figure 5). The pooling analysis showed that patients received PEG intervention had a lower incidence of pain compared with PRG and RIG (PEG versus PRG+RIG: 20.85% versus 41.26%; OR, 0.42; 95% CI, 0.27-0.67; *P* < 0.001) (Figure 4). And the same trends could be found when compared with PRG and RIG separately (PEG versus PRG: 19.34% versus 43.08%; OR, 0.49; 95% CI, 0.24-0.98; *P* < 0.05; PEG versus RIG: 31.60% versus 49.45%; OR, 0.42; 95% CI, 0.25-0.69; *P* < 0.001) (Figure 4).

### Sensitivity analysis, heterogeneity and bias

Sensitivity analysis was made in comparison with significant heterogeneity among studies (Table 3). The number of studies included in the comparisons of 30-day mortality, survival length, pain occurrence and success attempt rate were less than 10 studies. This indicated that the results were relatively unstable. The sensitivity analysis was not performed. Also, as there are less than 10 studies in allthe comparisons, a publicationbias assessment cannot be performed accurately.

## DISCUSSION

To the best of our knowledge, this meta-analysis is the first to assess the role of different gastrostomy approaches on ALS patients with nutritional difficulties. Our study has some interesting and useful findings: First, there were no differences in 30-day mortality and survival length after placement between PEG and FG;Second, PEG has a higher successful placement rate with low rate of post-procedural pain occurrence. This provides basis for the recommendation of PEG in the clinical practice.

ALS, a unique neurodegenerative disease, receives more and more attention with the prevalence of ice bucket challenge in recent years. Although tremendous efforts and money have been provided, it still has no cure [13]. The nutritional deficit is widely observed among ALS population, severely impairing the living conditions of them. Gastrostomy is a commonly applied method to solve this disorder. This approachis a useful predictor for the survival status of ALS patients. Several studies had established the efficacy of these approaches. Patients with gastrostomy are likely to survive a longer time than those refusing it [14]. There are two main methods widely used in the clinical practice: PEG and FG. The latter one includes RIG and PRG [5]. Both PEG and FG were widely used in the clinical practice of the disease, while whether there were any differences between them was still unknown. We further compared the improvement of survival status, and found that PEG had no difference on 30-day mortality and survival length, compared with FG or PRG and RIG, separately. They should be applied in the same place for the purpose of improving survival statues. But the limited number of studies may have made observation of significant associations difficult. More studies should be found in the future for thorough analysis of the survival improvement. Although researches have indicated that gastrostomy is a safe option for the sufferers [15], it also has some concurrent incidences or complications that we would not like to meet during and after the operation. Although we would like to analyze all the peri- and post-procedural complications, the insufficient data hindered our efforts. We only examined difficult procedure, success attempt rate, O_2_ desaturation, distress and respiratory disorder during procedure. Many patients could not receive the gastrostomy tube successfully, because of their intrinsic anatomical structure or concomitant problems. And we found a lower successful placement rate in patients with PEG than that receiving PRG and RIG. However, no such findings were drawn when FG was assessed separately. This indicated that the fluorescent assistance facilitated and the insertion process through a precise observation of anatomical structure. Then we didn't found any significance in the pooling analysis of difficult procedure, O_2_ desaturation, distress and respiratory disorder. There were many complaints of post-operational complications, including infection, granulation tissue, pain, anxiety, nausea, diahhoea, pneumonia, constipation, fatigue, tube replacement, aspiration, haemorrhage, mechanical obstructive and tube migration. In our study, PEG method was found to induce a lower rate of pain incidence than FG. But due to the limited number of studies in the comparison, this finding should be more cautious to get when assessing FG, separately (PRG and RIG). It might hinder the stability of the results. And the analysis of infection revealed no differences in all the comparisons.

Most of the studies included in the study were with high quality. These pooling analyses showed significant heterogeneity and the publication bias was unable to conclude due to the limited number of studies. Subgroup analysis were undertaken to indicate the credibility of our results. However, the number of the studies included was few. This might cause an ambiguity, which required more articles to be analyzed in the future. And the background information of the patients’ conditions in other systems or organs could not be got, and this should be investigated in the following clinical or meta-analysis study.

We intended to study more concurrent incidences or related complications, including economic expenses, nutritional improvements, infection incidence and so on, but the lack of raw data in these sections withdrew our attempt for a pooling analysis. We would make it in the following study to provide more interesting and meaningful findings on gastrostomy application in ALS patients.

In conclusion, PEG, PRG and RIG had their intrinsic advantages. The current evidences could not determine the preference of them. Further investigations should be done to reveal the most appropriate method for ALS patients.

## MATERIALS AND METHODS

### Systematic literature search

We used Preferred Reporting Items for Systematic Reviews and Meta-analysis (Supplementary Table 3) [16] and Meta-analysis of Observational Studies in Epidemiology (MOOSE) [17] recommendations for study reporting. The literature search was performed by 2 reviewers (Xiaolei Shi and Biying Yang) on articles published through to January 2017. A computerized search of the Pubmed, Web of Science, and Cochrane Library databases was performed without restriction on the language or publication type. Keywords and free text searches used the following keywords: “Percutaneous Endoscopic Gastrostomy” or “PEG” or “Fluoroscopic Gastrostomy” or “FG” or “Radiologically Inserted Gastrostomy” or “RIG” or “Per-oral Image-Guided Gastrostomy” or “PRG”, in combined with “Amyotrophic Lateral Sclerosis” or “ALS”. All reference sections of eligible studies and pertinent reviews were hand-reviewed for potential studies.

### Ethics, inclusion and exclusion criteria

All analyses were based on previously published studies; thus, ethical approval and patient consent were not required. To insure the homogeneity across studies, included studies should met the following criteria: (1) All available prospective or retrospective comparative studies (cohort studies, case-control studies or cross-sectional studies) that included any comparison of two or three of the three methods. (2) A ALS was strictly defined according to the El Escorial criteria [18]. (3) Patients were diagnosed with nutritional decline (BMI decline, weight loss, chewing, swallowing and eating difficulties) or aspiration risk. Exclusion criteria are as following: (1) Articles without the clear definition and description of ALS diagnosis criteria and gastrostomy indications were excluded; (2) review articles, case reports, editorials, letters to editorials and experimental studies were excluded.

### Selection and data extraction

Data from the included studies were extracted and summarized independently by the two authors (Xiaolei Shi and Biying Yang). Any disagreement was resolved by consensus-based discussion. Data including survival information (30-day mortality and survival time), peri- (difficult procedure, success attempt, O_2_ desaturation, distress, respiratory arrest, laryngeal spasm and haemorrhage) and post-procedural (infection, granulation tissue, pain, anxiety, nausea, diahhoea, pneumonia, constipation, fatigue, replacement, aspiration, haemorrhage and mechanical obstruction/tube migration) complications. The primary outcomes were the comparison of the data (30-day mortality, survival time, successful attempt rate, infection and pain) between PEG and FG (PRG and RIG). 30-day mortalityis defined as the mortality rate of patients 30 days after the placement of gastrostomy tube. Survival time is the living duration time since the operation procedure. Successful attempt rate means the success rate of tube insertion among patients with PEG and FG. Pain and infection occurrence means the incidence of them after the placement.

### Quality assessment and statistical analyses

The quality of observationalstudies was assessed by the Newcastle-Ottawa Quality Assessment Scale [19], which consists of three factors: Patient selection, comparability of the study groups, and assessment of outcome. A score of 0–9 was allocated to each study. Studies that achieved six or more stars were considered to be of high quality. The quality of cross-sectional studies was assessed by an 11-item checklist which was recommended by Agency for Healthcare Research and Quality (AHRQ) [20]. An item would be scored “0” if it was answered “NO” or “UNCLEAR”; if it was answered “YES”, then the item scored “*1*”. Article quality was assessed as follows: low quality = 0–3; moderate quality = 4–7; high quality = 8–11. Meta-analysis was performed on studies that provided data on outcomes of patients using the software package Revman 5. The subgroup analysis were undertaken in the comparison of these factors in PEG with PRG or RIG. The weighted mean difference (WMD) and odds ratio (OR) were used to compare continuous and dichotomous variables, respectively. All results were reported with 95% confidence intervals (CIs). Statistical heterogeneity was assessed using the *I*^2^ statistic with a Cochran *Q*-test, which describes the proportion of total variation that is attributable to differences among trials rather than sampling error (chance). An *I*^2^ value of <25% was defined to represent low heterogeneity, a value between 25% and 50% was defined as moderate heterogeneity and >50% was defined as high heterogeneity. The random-effects model was used if there was high heterogeneity between studies. Otherwise, the fixed-effects model was used. As there were less than 10 studies in this meta-analysis, we didn't do sensitivity analysis and publication bias.

## SUPPLEMENTARY MATERIALS FIGURES AND TABLES






