# Supplementary material for: Percutaneous endoscopic gastrostomy versus fluoroscopic gastrostomy in amyotrophic lateral sclerosis (ALS) sufferers with nutritional impairment: A meta-analysis of current studies
Source: Oncotarget. 2017 Nov 6;8(60):102244–53. doi: 10.18632/oncotarget.22288 (PMC5731950; doi:10.18632/oncotarget.22288)
Supplement: Supplementary file 1 [file oncotarget-08-102244-s001.pdf]

Percutaneous endoscopic gastrostomy versus fluoroscopic gastrostomy in amyotrophic lateral sclerosis (ALS) sufferers with nutritional impairment: A meta-analysis of current studies

SUPPLEMENTARY MATERIALS

Supplementary Table 1: Peri-procedural complications of each group

Study	Group	Difficult procedure	Success attempt	O_2 desaturation	Distress	Respiratory arrest	Laryngeal spasm	Haemorrhage
Thornton	PEG	9 in 20[a]	11 in 20[a]	NA	NA	1	NA	NA
	PRG	8 in 25[a]	25 in 25[a]	NA	NA	0	NA	NA
A Chio	PEG	NA	23	NA	NA	NA	NA	NA
	PRG	NA	25	NA	NA	NA	NA	NA
Desport	PEG	NA	NA	NA	NA	NA	NA	NA
	RIG	NA	NA	NA	NA	NA	NA	NA
Shaw	PEG	NA	NA	NA	NA	NA	NA	NA
	RIG	NA	NA	NA	NA	NA	NA	NA
Blondet	PEG	NA	15	NA	NA	NA	NA	NA
	PRG	NA	22	NA	NA	NA	NA	NA
Allen	PEG	2	48	2	0	4	NA	NA
	RIG	1	50	1	1	1	NA	NA
ProGas Study	PEG	26	160 in 171[a]	6	26	0	2	0
Group	RIG	13	118 in 125[a]	2	4	0	1	3
	PRG	7	42 in 45[a]	3	2	0	0	0

PEG, Percutaneous Endoscopic Gastrostomy; PRG, Per-oral Image-Guided Gastrostomy; RIG, Radiologically Inserted Gastrostomy; NA, Not Applicable. [a]means the incidence number in total events.

Supplementary Table 2: Post-procedural complications of each group. See Supplementary_Table_2

Supplementary Table 3: PRISMA checklist. See Supplementary_Table_3

	PEG		PRG			Odds Ratio
Study or Subgroup	Events	Total	Events	Total	Weight	M-H, Fixed, 95% CI
Thorton 2002	9	20	8	25	29.6%	1.74 [0.51, 5.87]
ProGas Study Group 2015	26	163	7	43	70.4%	0.98 [0.39, 2.43]
**Total (95% CI)**		**183**		**68**	**100.0%**	**1.20 [0.58, 2.51]**
Total events	35		15			
Heterogeneity: Chi² = 0.55, df = 1 (P = 0.46); I² = 0%						
Test for overall effect: Z = 0.49 (P = 0.62)						
Allen 2013	2	57	1	51	7.5%	1.82 [0.16, 20.67]
ProGas Study Group 2015	26	163	13	121	92.5%	1.58 [0.77, 3.21]
**Total (95% CI)**		**220**		**172**	**100.0%**	**1.59 [0.81, 3.16]**
Total events	28		14			
Heterogeneity: Chi² = 0.01, df = 1 (P = 0.91); I² = 0%						
Test for overall effect: Z = 1.34 (P = 0.18)						
Thorton 2002	9	20	8	25	18.0%	1.74 [0.51, 5.87]
Allen 2013	2	57	1	51	4.7%	1.82 [0.16, 20.67]
ProGas Study Group 2015	26	163	20	164	77.3%	1.37 [0.73, 2.56]
**Total (95% CI)**		**240**		**240**	**100.0%**	**1.45 [0.84, 2.51]**
Total events	37		29			
Heterogeneity: Chi² = 0.15, df = 2 (P = 0.93); I² = 0%						
Test for overall effect: Z = 1.35 (P = 0.18)						

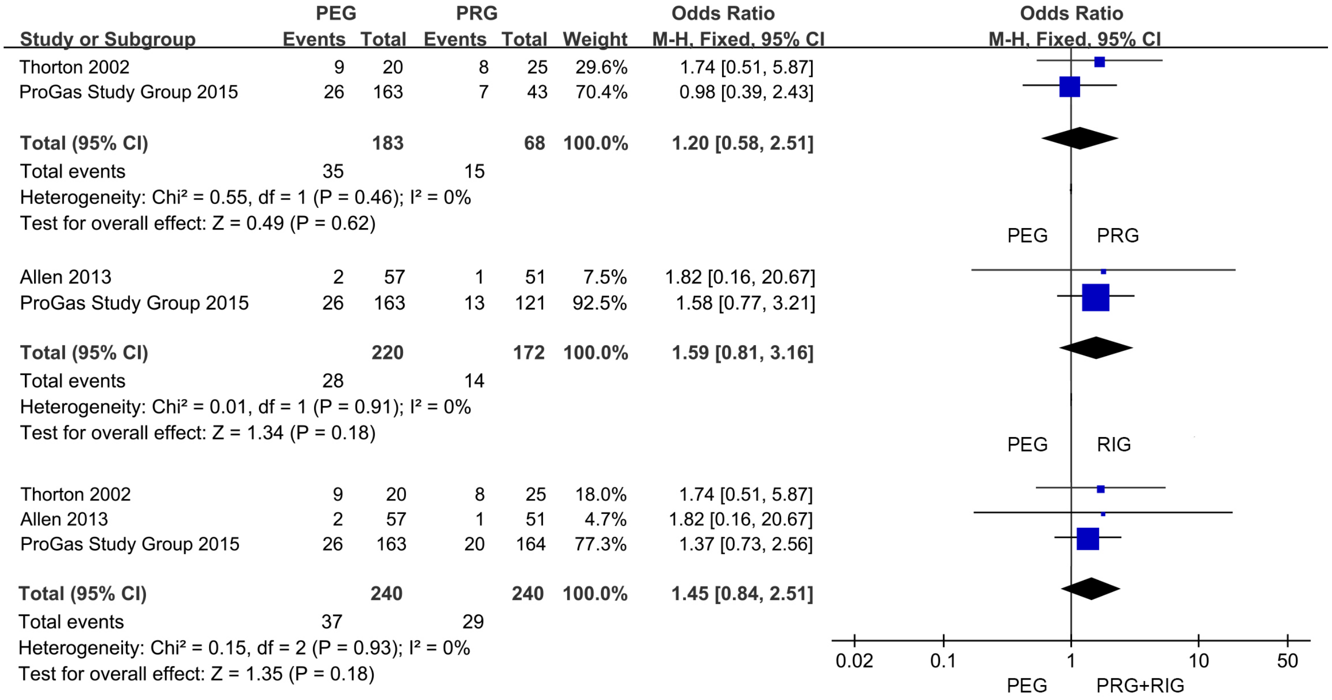

Supplementary Figure 1: Forest plot and meta-analysis of difficult procedure.

	PEG		RIG			Odds Ratio
Study or Subgroup	Events	Total	Events	Total	Weight	M-H, Fixed, 95% CI
Allen 2013	2	57	1	51	31.5%	1.82 [0.16, 20.67]
ProGas Study Group 2015	6	163	2	121	68.5%	2.27 [0.45, 11.47]
**Total (95% CI)**		**220**		**172**	**100.0%**	**2.13 [0.56, 8.17]**
Total events	8		3			
Heterogeneity: Chi² = 0.02, df = 1 (P = 0.88); I² = 0%						
Test for overall effect: Z = 1.10 (P = 0.27)						
Allen 2013	2	57	1	51	17.5%	1.82 [0.16, 20.67]
ProGas Study Group 2015	6	163	5	164	82.5%	1.22 [0.36, 4.06]
**Total (95% CI)**		**220**		**215**	**100.0%**	**1.32 [0.45, 3.88]**
Total events	8		6			
Heterogeneity: Chi² = 0.08, df = 1 (P = 0.77); I² = 0%						
Test for overall effect: Z = 0.51 (P = 0.61)						

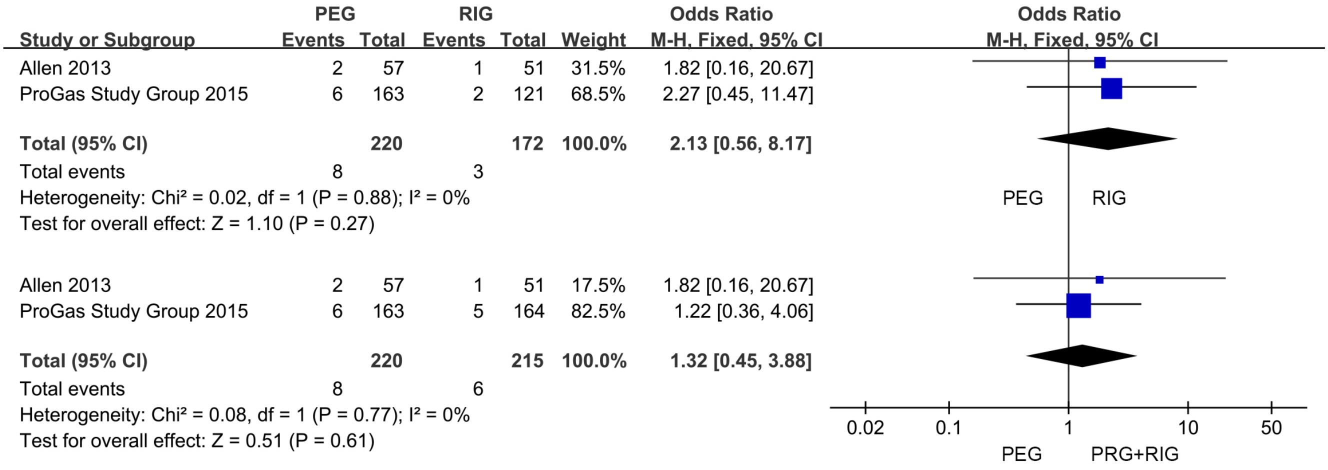

Supplementary Figure 2: Forest plot and meta-analysis of oxygen desaturation.

Study or Subgroup	PEG Events	PEG Total	RIG Events	RIG Total	Weight	Odds Ratio M-H, Random, 95% CI
Allen 2013	0	57	1	51	36.2%	0.29 [0.01, 7.35]
ProGas Study Group 2015	26	163	4	121	63.8%	5.55 [1.88, 16.37]
**Total (95% CI)**		**220**		**172**	**100.0%**	**1.92 [0.12, 30.63]**
Total events	26		5			
Heterogeneity: Tau² = 2.83; Chi² = 2.88, df = 1 (P = 0.09); I² = 65%						
Test for overall effect: Z = 0.46 (P = 0.65)						
Allen 2013	0	57	1	51	34.6%	0.29 [0.01, 7.35]
ProGas Study Group 2015	26	163	6	164	65.4%	5.00 [2.00, 12.50]
**Total (95% CI)**		**220**		**215**	**100.0%**	**1.87 [0.13, 26.48]**
Total events	26		7			
Heterogeneity: Tau² = 2.58; Chi² = 2.76, df = 1 (P = 0.10); I² = 64%						
Test for overall effect: Z = 0.46 (P = 0.64)						

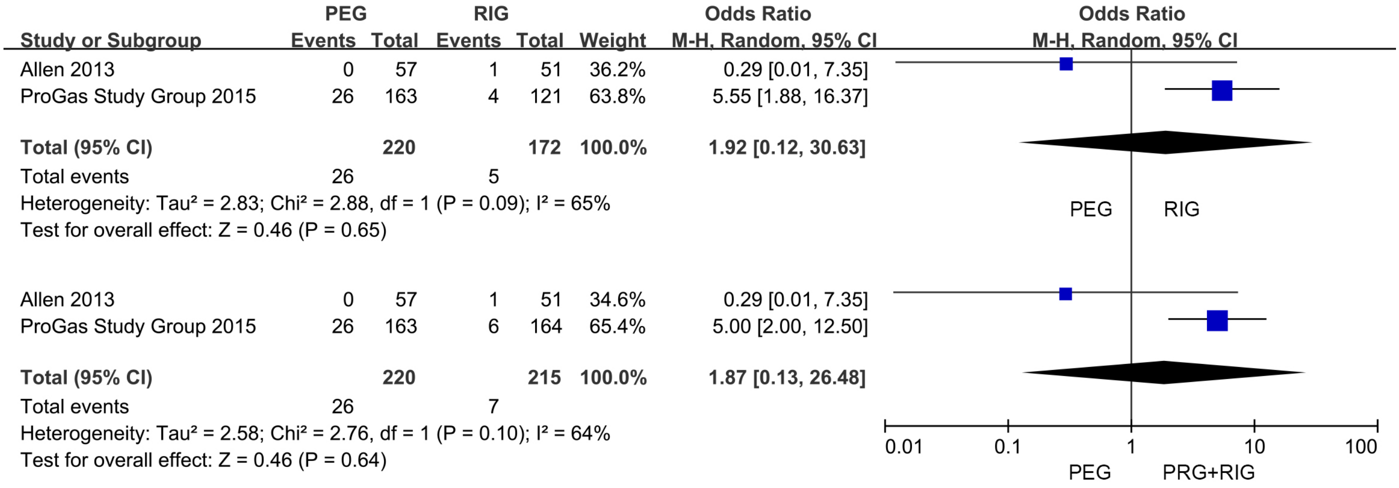

Supplementary Figure 3: Forest plot and meta-analysis of distress occurrence.

Study or Subgroup	PEG Events	PEG Total	PRG Events	PRG Total	Weight	Odds Ratio M-H, Fixed, 95% CI
Thorton 2002	1	11	0	25	100.0%	7.29 [0.27, 193.68]
ProGas Study Group 2015	0	163	0	43		Not estimable
**Total (95% CI)**		**174**		**68**	**100.0%**	**7.29 [0.27, 193.68]**
Total events	1		0			
Heterogeneity: Not applicable						
Test for overall effect: Z = 1.19 (P = 0.24)						
Allen 2013	4	57	1	51	100.0%	3.77 [0.41, 34.92]
ProGas Study Group 2015	0	163	0	164		Not estimable
**Total (95% CI)**		**220**		**215**	**100.0%**	**3.77 [0.41, 34.92]**
Total events	4		1			
Heterogeneity: Not applicable						
Test for overall effect: Z = 1.17 (P = 0.24)						
Thorton 2002	1	11	0	25	22.0%	7.29 [0.27, 193.68]
Allen 2013	4	57	1	51	78.0%	3.77 [0.41, 34.92]
ProGas Study Group 2015	0	163	0	164		Not estimable
**Total (95% CI)**		**231**		**240**	**100.0%**	**4.55 [0.71, 29.04]**
Total events	5		1			
Heterogeneity: Chi² = 0.11, df = 1 (P = 0.74); I² = 0%						
Test for overall effect: Z = 1.60 (P = 0.11)						

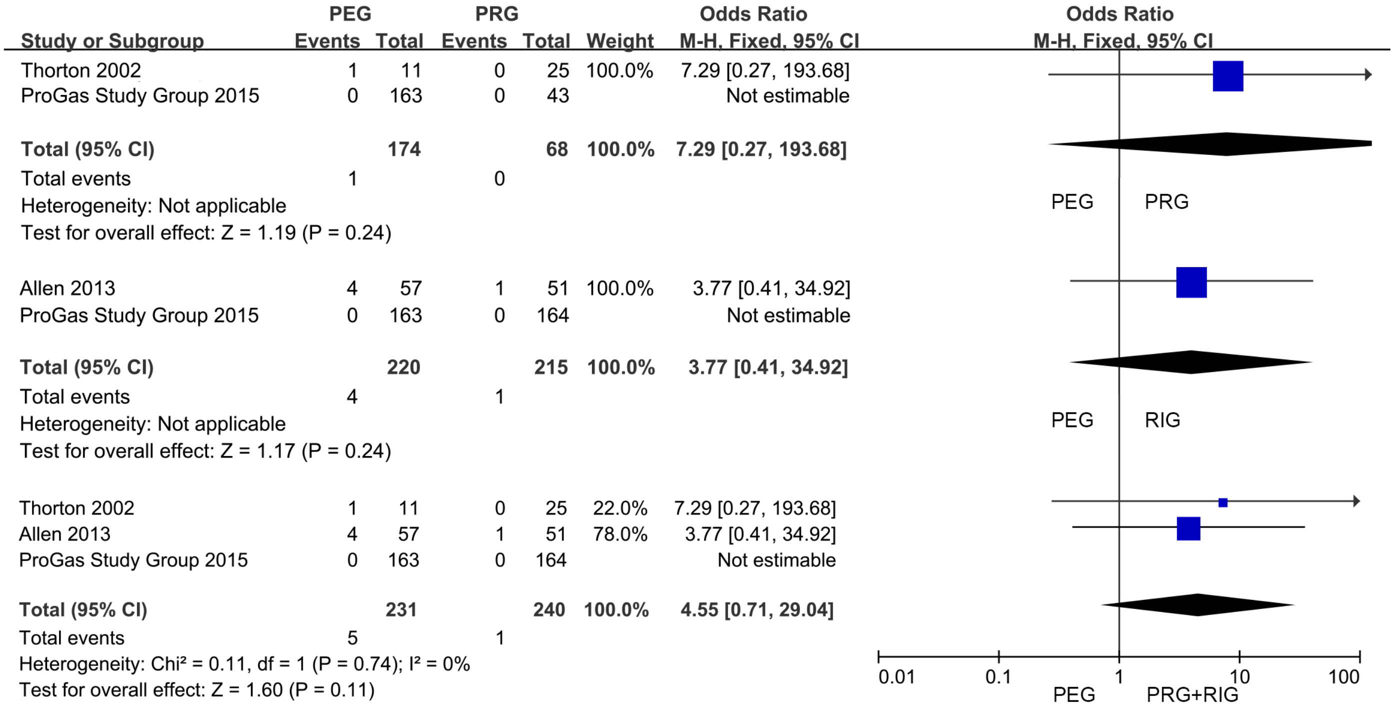

Supplementary Figure 4: Forest plot and meta-analysis of respiratory arrest.

Study or Subgroup	PEG Events	PEG Total	PRG Events	PRG Total	Weight	Odds Ratio M-H, Fixed, 95% CI
Thorton 2002	20	163	3	43	93.8%	1.86 [0.53, 6.59]
ProGas Study Group 2015	1	11	0	25	6.2%	7.29 [0.27, 193.68]
**Total (95% CI)**		**174**		**68**	**100.0%**	**2.20 [0.67, 7.19]**
Total events	21		3			
Heterogeneity: Chi² = 0.58, df = 1 (P = 0.45); I² = 0%						
Test for overall effect: Z = 1.31 (P = 0.19)						
Thorton 2002	20	163	24	164	98.7%	0.82 [0.43, 1.54]
ProGas Study Group 2015	1	11	0	25	1.3%	7.29 [0.27, 193.68]
**Total (95% CI)**		**174**		**189**	**100.0%**	**0.90 [0.49, 1.67]**
Total events	21		24			
Heterogeneity: Chi² = 1.65, df = 1 (P = 0.20); I² = 39%						
Test for overall effect: Z = 0.33 (P = 0.74)						

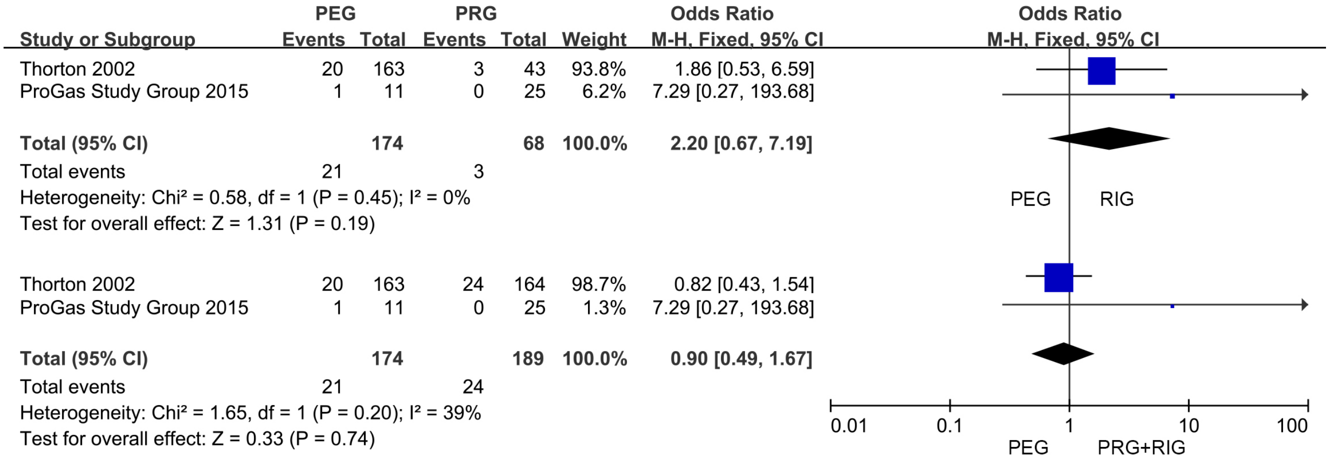

Supplementary Figure 5: Forest plot and meta-analysis of infection rate.
