# Supplementary material for: Percutaneous endoscopic gastrostomy versus fluoroscopic gastrostomy in amyotrophic lateral sclerosis (ALS) sufferers with nutritional impairment: A meta-analysis of current studies
Source: Oncotarget. 2017 Nov 6;8(60):102244–53. doi: 10.18632/oncotarget.22288 (PMC5731950; doi:10.18632/oncotarget.22288)
Supplement: Supplementary file 2 [file oncotarget-08-102244-s002.docx]

**Supplementary Table 2: Post-procedural complications of each group**

| **Study** | **Group** | **Infection** | **Granulation tissue** | **Pain** | **Anxiety** | **Nausea** | **Diahhoea** |
| --- | --- | --- | --- | --- | --- | --- | --- |
| Thornton | PEG | 1 | NA | NA | NA | NA | NA |
|  | PRG | 0 | NA | NA | NA | NA | NA |
| Chio | PEG | NA | NA | NA | NA | NA | NA |
|  | PRG | NA | NA | NA | NA | NA | NA |
| Desport | PEG | NA | NA | NA | NA | NA | NA |
|  | RIG | NA | NA | NA | NA | NA | NA |
| Shaw | PEG | NA | NA | NA | NA | NA | NA |
|  | RIG | NA | NA | NA | NA | NA | NA |
| Blondet | PEG | NA | NA | NA | NA | NA | NA |
|  | PRG | NA | NA | NA | NA | NA | NA |
| Allen | PEG | NA | NA | NA | NA | NA | NA |
|  | RIG | NA | NA | NA | NA | NA | NA |
| ProGas | PEG | 20 | 15 | 25 | 10 | 12 | 6 |
| Study | RIG | 21 | 19 | 34 | 24 | 10 | 10 |
| Group | PRG | 3 | 3 | 10 | 1 | 2 | 3 |

(To be continued)

| **Pneumonia** | **Constipation** | **Fatigue** | **Replacement** | **Aspiration** | **Haemorrhage** | **Mechanical obstructive or tube migration** |
| --- | --- | --- | --- | --- | --- | --- |
| NA | NA | NA | 2 in 11^a^ | NA | NA | NA |
| NA | NA | NA | 17 in 10^a^ | NA | NA | NA |
| NA | NA | NA | NA | NA | NA | NA |
| NA | NA | NA | NA | NA | NA | NA |
| NA | NA | NA | NA | NA | 0 | NA |
| NA | NA | NA | NA | NA | NA | NA |
| NA | NA | NA | NA | NA | NA | NA |
| NA | NA | NA | NA | NA | NA | NA |
| NA | NA | NA | NA | NA | NA | NA |
| NA | NA | NA | NA | NA | NA | NA |
| NA | NA | NA | NA | NA | NA | NA |
| NA | NA | NA | NA | NA | NA | NA |
| 4 | 16 | 15 | NA | NA | NA | NA |
| 4 | 22 | 23 | NA | NA | NA | NA |
| 4 | 9 | 4 | NA | NA | NA | NA |

PEG, Percutaneous Endoscopic Gastrostomy; PRG, Per-oral Image-Guided Gastrostomy; RIG, Radiologically Inserted Gastrostomy; NA, Not Applicable.^a^ means the incidence number in total events.
